# Quantitative Proteomics Analysis Reveals That Cyclooxygenase-2 Modulates Mitochondrial Respiratory Chain Complex IV in Cardiomyocytes

**DOI:** 10.3390/ijms232113476

**Published:** 2022-11-03

**Authors:** Maria Soledad Alvarez, Estefanía Núñez, Marina Fuertes-Agudo, Carme Cucarella, Maria Fernandez-Velasco, Lisardo Boscá, Jesús Vázquez, Rodrigue Rossignol, Paloma Martin-Sanz, Marta Casado

**Affiliations:** 1 Instituto de Biomedicina de Valencia (IBV), CSIC, Jaume Roig 11, 46010 Valencia, Spain; 2 Laboratory of Cardiovascular Proteomics, Centro Nacional de Investigaciones Cardiovasculares (CNIC), Melchor Fernández Almagro, 3, 28029 Madrid, Spain; 3 CIBER de Enfermedades Cardiovasculares (CIBERCV), Monforte de Lemos 3-5, 28029 Madrid, Spain; 4 Centro de Investigación Biomédica en Red de Enfermedades Hepáticas y Digestivas (CIBERehd), Monforte de Lemos 3-5, 28029 Madrid, Spain; 5 Instituto de Investigación Hospital Universitario La Paz, IDIPAZ, Paseo de la Castellana 261, 28046 Madrid, Spain; 6 Instituto de Investigaciones Biomedicas Alberto Sols (IIBM), CSIC-UAM, Arturo Duperier 4, 28029 Madrid, Spain; 7 Laboratoire Maladies Rares, CHU Pellegrin Place Amelie Rab, 33076 Bordeaux, France

**Keywords:** COX-2, prostaglandins, mitochondria, respiratory capacity, transgenic animals

## Abstract

The biochemical mechanisms of cell injury and myocardial cell death after myocardial infarction remain unresolved. Cyclooxygenase 2 (COX-2), a key enzyme in prostanoid synthesis, is expressed in human ischemic myocardium and dilated cardiomyopathy, but it is absent in healthy hearts. To assess the role of COX-2 in cardiovascular physiopathology, we developed transgenic mice that constitutively express functional human COX-2 in cardiomyocytes under the control of the α-myosin heavy chain promoter. These animals had no apparent phenotype but were protected against ischemia-reperfusion injury in isolated hearts, with enhanced functional recovery and diminished cellular necrosis. To further explore the phenotype of this animal model, we carried out a differential proteome analysis of wild-type vs. transgenic cardiomyocytes. The results revealed a tissue-specific proteomic profile dominated by mitochondrial proteins. In particular, an increased expression of respiratory chain complex IV proteins was observed. This correlated with increased catalytic activity, enhanced respiratory capacity, and increased ATP levels in the heart of COX-2 transgenic mice. These data suggest a new link between COX-2 and mitochondria, which might contribute to the protective cardiac effects of COX-2 against ischemia-reperfusion injury.

## 1. Introduction

The World Health Organization has reported that ischemic heart disease and stroke are the leading cause of death throughout the world [1]. In patients with acute myocardial infarction (MI), the treatment of choice to reduce acute myocardial ischemic injury and limit MI size is timely and effective myocardial reperfusion. However, reperfusion causes additional damage to the affected myocardium [2]. Myocardial ischemia-reperfusion (I/R) injury (IRI) causes different pathological events such as arrhythmias, myocardial stunning and hibernation or microvascular damage [3]. Several effectors have been recognized as key mediators in IRI, such as oxidative stress, intracellular calcium overload, neutrophil activation, or excessive intracellular osmotic load [4].

Prostaglandin H synthase (prostaglandin G/H synthase, EC 1.14.99.1), also known as cyclooxygenase (COX), catalyzes the rate-limiting step in the synthesis of prostaglandins (PGs) and thromboxanes converting arachidonic acid into PGH_2_, which serves as a substrate for different cell-specific prostaglandin and thromboxane synthases. Two COX isoenzymes have been identified. The COX-1 isoform is ubiquitous and constitutively expressed in a wide variety of tissues and is responsible for the low but constant PG synthesis required for tissue homeostasis [5]. Meanwhile, the COX-2 isoform is undetectable in most tissues, except the placenta, testis, macula densa, and brain. A variety of stimuli (inflammation, growth factors, hormones, reactive oxygen intermediates, and oncogenes) can rapidly induce COX-2 in several cell types [6]. COX-2 is expressed in human ischemic myocardium and dilated cardiomyopathy but is absent in a normal heart [7]. Moreover, Goren et al. showed that whereas fetal cardiomyocytes express COX-2 after LPS and pro-inflammatory challenge, neonatal and adult cardiomyocytes are unable to express the enzyme except after a prolonged period of cell culture. These results suggest that COX-2 expression is restricted to those situations in which dedifferentiation or proliferation occurs [8]. Additionally, Saito et al. showed that COX-2 inhibition improves cardiac function after MI in human or animal models [9]. These results point to the harmful role of COX-2 in the cardiovascular system. However, Bolli et al. have demonstrated that ischemic preconditioning (PC), a phenomenon in which brief episodes of ischemia and reperfusion protect the heart from a subsequent prolonged ischemic period, upregulates the expression and activity of COX-2 and NOS-2 in heart, mediating the protective effects of the late phase of PC [10]. Furthermore, COX-2 knockout animals show a greater IRI [11].

To clarify the role of COX-2 in the heart, we developed transgenic mice that constitutively express functional human COX-2 (*hCOX-2-Tg*) in cardiomyocytes under the control of the α-myosin heavy chain promoter on a B6D2/JRccHsd background (B6D2-Tg (MHC-*PTGS2*)17/Upme). Our previous studies revealed that these animals were protected against IRI in isolated hearts, with enhanced recovery of function and diminished cellular necrosis. Pretreatment of these animals with the specific COX-2 inhibitor 5-dimethyl-3(3-fluorophenyl)-4-(4-methylsulfonyl) phenyl-2(5H)-furanone (DFU) reduced the cardioprotective status, with a negative correlation between myocardial PGE_2_ levels and the extent of cell death [12].

As a first step to shedding on the mechanisms whereby COX-2 induces cardioprotection, in this study, we analyzed the differential proteome of cardiomyocytes from wild-type (*Wt*) vs. *hCOX-2-Tg* animals under basal conditions, identifying a list of up- and downregulated proteins. After clustering these proteins according to their biological functions, we observed that many of them are mitochondrial proteins. In agreement with these results, oxygen consumption, cytochrome c oxidase activity, and ATP levels were increased in the heart of *hCOX-2-Tg* mice. Taken together, these data show a relationship between COX-2 and mitochondria that might explain the protective effect of COX-2 expression against IRI.

## 2. Results

### 2.1. Proteome Differences in Cardiomyocytes from hCOX-2-Tg Mice

Our previous results showed that transgenic COX-2 expression induces protection against ischemia-reperfusion [12]. To gain insight into the role of COX-2 in the heart, we performed a quantitative proteomics approach to identify differentially expressed proteins between cardiomyocytes from *Wt* and *hCOX-2 Tg* mice under basal conditions. Using a strict 5% FDR criterion, 22 upregulated and 6 downregulated proteins were found (Table 1). The profile of protein changes was dominated by mitochondrial proteins, whose abundance was systematically increased in *hCOX-2 Tg* mice.

**Figure 1:**
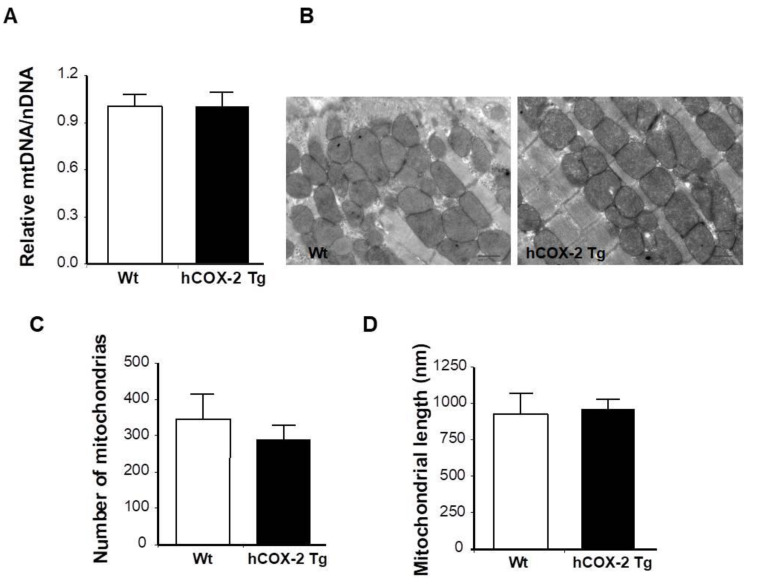
Mitochondrial biogenesis in *hCOX-2 Tg* mice. (**A**) Mitochondrial-to-nuclear DNA ratio was determined in hearts from *Wt* (*n* = 13) and *hCOX-2 Tg* (*n* = 16) mice by real-time PCR. Data are expressed relative to *Wt* animals. (**B**) Transmission electron microscopic micrographs of hearts from *Wt* or *hCOX-2 Tg* mice. Original magnification ×6000. Mitochondria density (**C**) and size (**D**) were analyzed. Data are means ± SD, *n* = 15 fields per heart.

Since the observed increase in mitochondrial proteins might reflect an increase in total cellular mitochondrial quantity, we determined mitochondrial biogenesis by measuring mitochondrial DNA content. As shown in Figure 1A, mitochondrial DNA was not affected by COX-2 expression. Mitochondrial biogenesis was also assessed by electron microscopy. No ultrastructural differences were observed in cardiac samples between *hCOX-2-Tg* and *Wt* mice (Figure 1B). Furthermore, quantitative analysis revealed that cardiomyocyte-specific COX-2 expression did not modify either the density or the size of mitochondria in the hearts of *hCOX-2-Tg* mice (Figure 1C,D).

**Figure 2:**
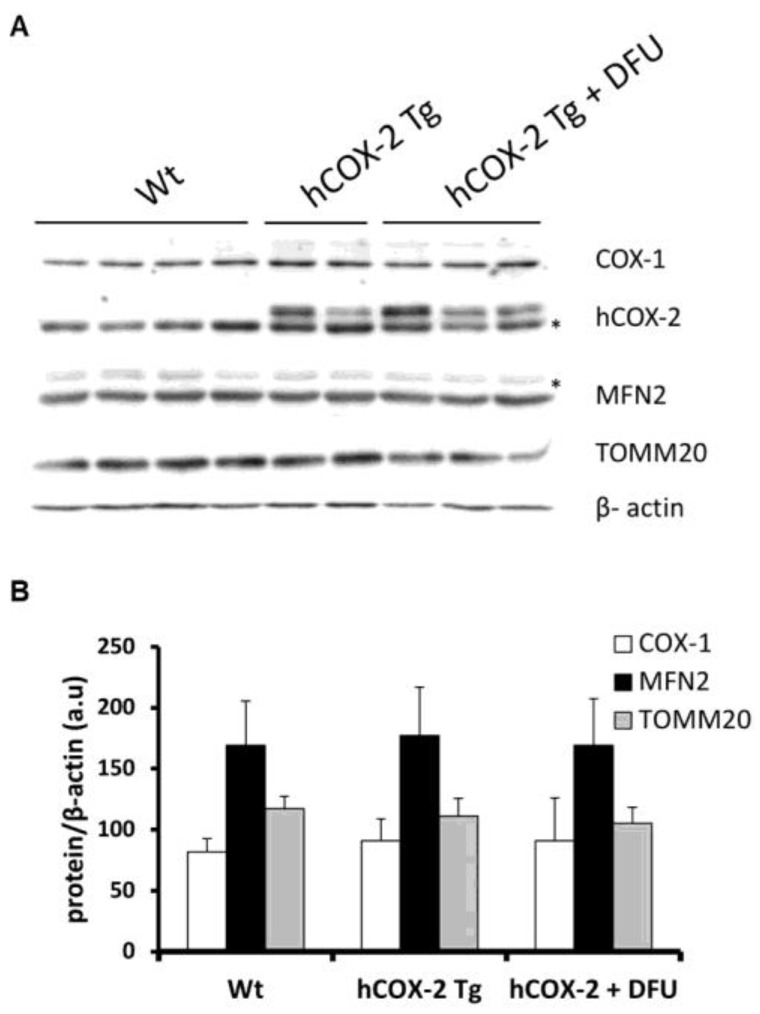
Mitochondrial and cytosolic protein expression in *hCOX-2 Tg* mice. Total extracts were prepared from *Wt* and *hCOX-2 Tg* mice hearts, and proteins (30 μg/lane) were analyzed by Western blot. (**A**) Representative image showing the expression of COX-1, human COX-2, and proteins located at cytosol (β-actin) or mitochondria (MFN2 and TOMM20). * nonspecific bands. (**B**) For densitometric analysis, the bands were scanned and band densities were quantified using Image J 1.50a software. Relative band intensity of COX-1, MFN2, and TOMM20 normalized to that β-actin are presented as means ± SD of 2 independent immunoblots.

Finally, and consistent with unaltered mitochondrial biogenesis, the ratio of TOMM20, a mitochondrial protein, to β-actin, a cytosolic protein, was also unaffected, reinforcing the idea that mitochondrial number is not disturbed. Moreover, the expression of mitofusin-2 (MFN2) was not altered by COX-2 expression consistent with no alteration in mitochondrial morphology (Figure 2). Furthermore, DFU treatment, a specific COX-2 inhibitor, also showed no changes in the expression of the proteins analyzed, confirming that COX-2 did not affect mitochondrial dynamics.

### 2.2. Canonical Pathways and Protein Network Analysis Altered in hCOX-2 Tg Animals

**Figure 3:**
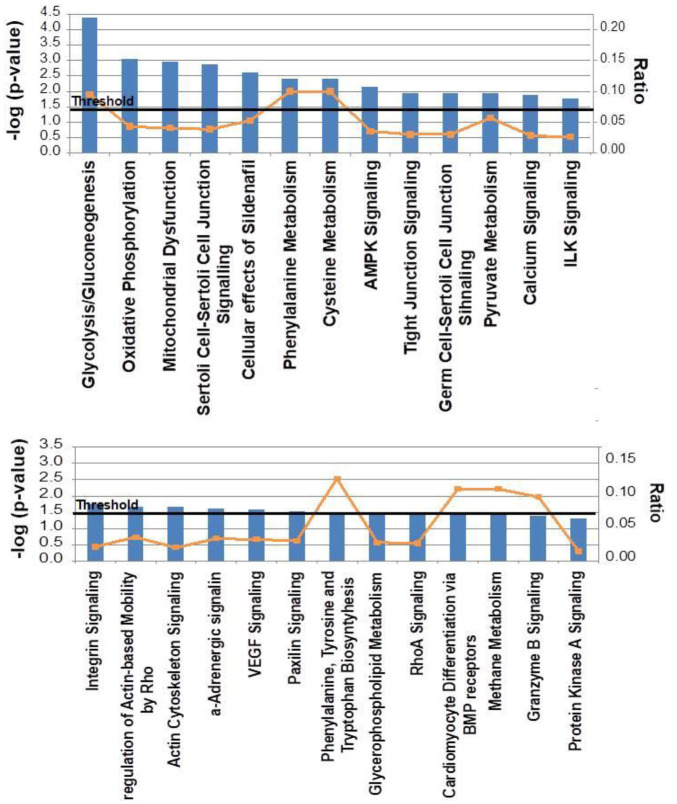
Canonical pathways significantly regulated in *hCOX-2 Tg* mice. Bioinformatic analyses were performed using IPA software. The most statistically significant canonical pathways are listed according to their *p* value (−log) (blue bars) and the ratio of the list of genes found in each pathway over the total number of genes in that pathway (orange squares). The threshold line corresponds to a *p* value of 0.05.

**Figure 4:**
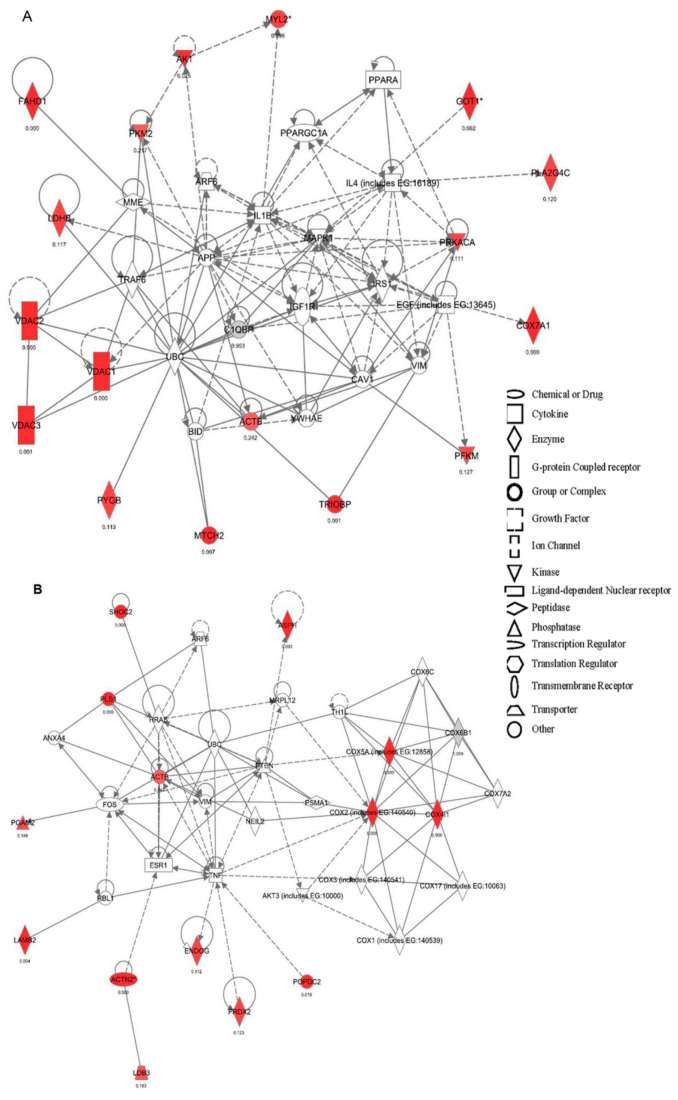
Networks associated with cardiac human COX-2 constitutive expression identified by ingenuity pathway analysis. Red symbols indicate upregulation, whereas green symbols indicate downregulated proteins. Gray symbols indicate a regulation that did not reach statistical significance. Proteins indicated in white are those available in the IPA database but not detected in the present study. The shapes of the symbols denote the molecular class of the proteins as defined in the legends. Solid lines indicate direct molecular interactions, whereas dashed lines indicate indirect molecular interactions. Network 1 (**A**) included FAHD1, PKM2, AK1, MYL2, GOT1, PLA2G4C, LDBH, PRKACA, VDAC1-3, ACTB, COX7A1, and PFKM s, whereas Network 2 (**B**) included SHOC2, ASPH, PGAMS, ACTB, COX5A, COX2, COX4I1, LAMB2, ACTN2, ENDOG, PRDX2, and POPDC2 as differentially expressed proteins.

To gain insight into the biological significance of quantitative proteomic results, we performed an enrichment analysis using the Ingenuity Pathway Analysis (IPA) software. The highest ranked canonical pathways that were affected were glycolysis/gluconeogenesis, oxidative phosphorylation, and mitochondrial dysfunction (Figure 3). The IPA analysis also identified two protein networks that were statistically significant (*p* < 0.05). Network 1 obtained the highest significant score (32; Figure 4A). The main function of this network was associated with carbohydrate metabolism, cardiovascular disease, or tissue morphology, whereas Network 2, with a significant score of 25, was associated with cell morphology, cell function and maintenance, and cell death (Figure 4B).

**Figure 5:**
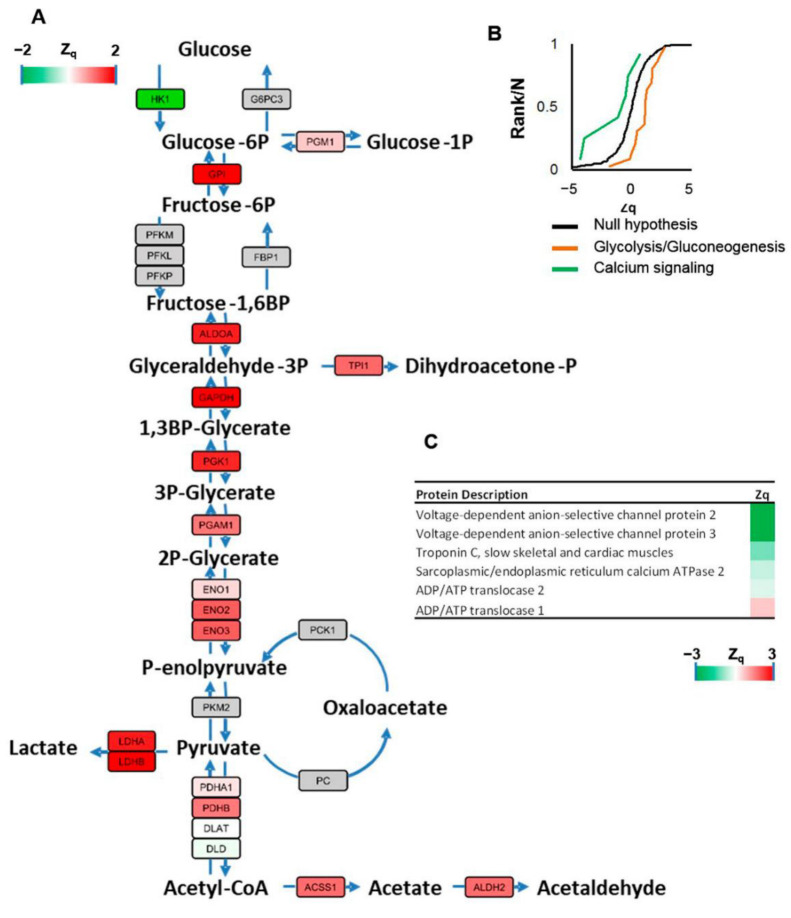
Systems biology analysis of quantified proteins in *hCOX-2 Tg* mice. (**A**) Representative image of the enzymes involved in glycolysis and gluconeogenesis. All enzymes quantified in this pathway were downregulated (red color), except for HK1, which was increased (green color). Gray color indicates enzymes that were not quantified. (**B**) Cumulative distributions of the standardized variable at the protein level (i.e., corrected log2 ratios of proteins expressed in units of standard deviations), for proteins belonging to glycolysis/gluconeogenesis and calcium signaling. The black sigmoid is the theoretical null hypothesis distribution; a displacement toward the left indicates an increase in protein concentration in *hCOX2 Tg* mice. (**C**) Calcium signaling proteins quantified in *hCOX-2 Tg* mice.

To reinforce the results obtained in the analysis of altered proteins, we performed a threshold-free functional analysis of coordinated protein responses using the SBT model [14]. We found that the glycolysis/gluconeogenesis pathways were downregulated in *h-COX-2 Tg* mice. A close inspection revealed that most enzymes of the glycolysis pathway were decreased (Figure 5A,B). Furthermore, the calcium signaling pathway was increased by the constitutive expression of COX-2 in the cardiomyocytes (Figure 5B), while the two mitochondrial VDAC proteins were responsible for the upregulation of calcium signaling (Figure 5C).

### 2.3. Oxygen Consumption Rate and ATP Generation Are Elevated in Heart Mitochondria from hCOX-2 Tg Mice

**Figure 6:**
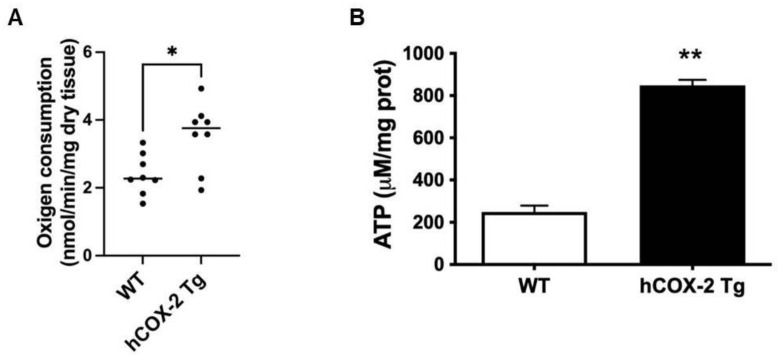
Oxygen consumption and ATP levels are elevated in *hCOX-2 Tg* mice. (**A**) Oxygen consumption of permeabilized fibers from *Wt* and *hCOX-2 Tg* mice was measured as described in Material and Methods. Results are means ± SD; *n* = 3; * *p* < 0.05 between animal models. (**B**) ATP levels were determined in a pool of tissue homogenates (*n* = 4) from *Wt* and *hCOX-2 Tg* mice. ** *p* < 0.01.

As shown above, the proteomic profile of *hCOX-2 Tg* cardiomyocytes was dominated by changes in proteins related to the mitochondrial oxidative phosphorylation pathway (OxPhos). To study whether this variation in the proteome translated into altered mitochondrial function in *hCOX-2 Tg* hearts, we measured oxygen consumption and ATP levels in permeabilized cardiac fibers or frozen hearts, respectively, from *Wt* and *hCOX-2 Tg* animals. As shown in Figure 6A, we observed a significant increase in respiratory capacity in *hCOX-2 Tg* compared to *Wt* mice. We also quantified ATP production in both groups using a bioluminometric assay. A significant difference in total cellular ATP content was seen at baseline between the genotypes (Figure 6B).

### 2.4. Constitutive COX-2 Expression in Cardiomyocytes Increases the Proportion of Complex IV Proteins

**Figure 7:**
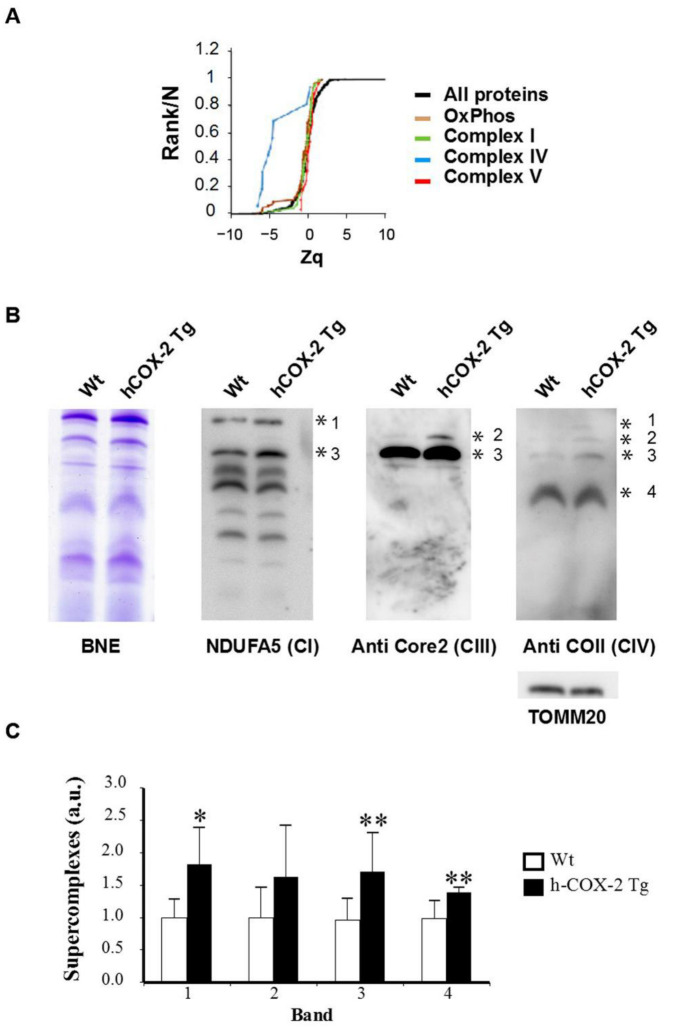
Analysis of mitochondrial supercomplex components. (**A**) Cumulative distributions of the standardized variable at the protein level (i.e., corrected log_2_ ratios of proteins expressed in standard deviation units), for all OxPhos proteins or those belonging to Complex I, III, IV and V. The black sigmoid is the theoretical null hypothesis distribution; a shift to the left indicates an increase in protein abundance in *hCOX2 Tg* mice. (**B**) Representative image of the detection of the respiratory complexes and potential supercomplexes after blue native gel electrophoresis analysis from digitonin solubilized mitochondria from *Wt* and *h-COX-2 Tg* mice hearts, followed by Western blot analysis using sequentially specific antibodies against complexes I, III, and IV. (**C**) Densitometric analysis of the expression of complexes. Data were normalized by calculating the ratios of complexes and TOMM20 band densities, expressed in arbitrary units. Data represent the mean values ± SD. * *p* < 0.05; ** *p* < 0.01 *hCOX-2 Tg* mice vs. *Wt*.

After observing increased routine respiration in *hCOX-2 Tg* mice, we specifically analyzed the proteins of the electron transport chain (ETC). We found that many proteins of complex IV were significantly increased while those of the other complexes were unaffected (Figure 7A). Since ETC is organized as a branched chain of four electron transfer chain complexes (I to IV) and ATP synthase (complex V), we analyzed whether any of these complexes were selectively affected by COX-2 expression. Furthermore, OxPhos complexes are known to assemble into supramolecular structures (supercomplexes, SC) to optimize their function [15]; thus, we assessed the effect of COX-2 on the assembly of SC by BNGE of digitonin-treated mitochondria. We were able to confirm the presence of SC I-IV (band 1), III-IV (band 2), the respirasome (I-III-IV, band 3), and complex IV (band 4) (Figure 7B). The amount of complex IV in the different bands was significantly increased in *hCOX-2 Tg* hearts (Figure 7C), validating the proteomics results.

**Figure 8:**
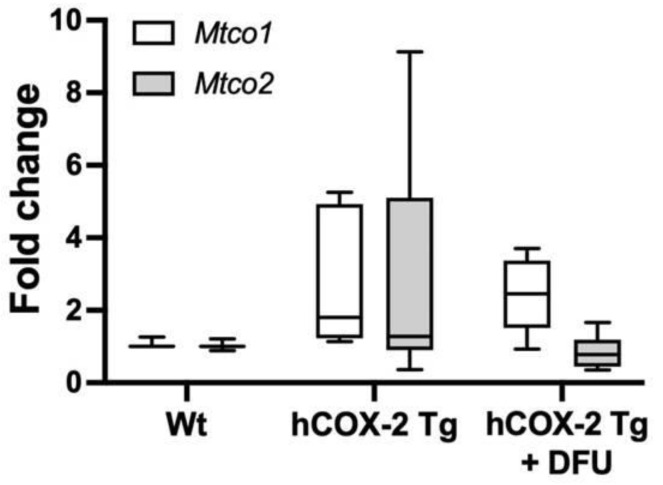
Gene expression of complex IV subunits is not modified in *hCOX-2 Tg* mice. Quantities of *Mtco1* and *Mtco2* mRNAs (the R_0_ values) were determined in hearts from *Wt* (*n* = 25), *hCOX-2 Tg* (*n* = 20), and *hCOX-2 Tg* mice treated with the COX-2 inhibitor DFU (*hCOX-2 Tg*+DFU, *n* = 13) using LinRegPCR data analysis. The amount of mRNA was then divided by the R_0_ value of *Gapdh* as normalization. Data are expressed as fold change relative to *Wt* mice.

Afterward, we analyzed the transcriptional level for CIV subunits 1 and 2 (both increased in proteomic analysis. See Table 1). mRNA levels were not affected by COX-2 expression, pointing to a post-transcriptional regulatory mechanism (Figure 8).

### 2.5. Complex IV Protein Enrichment Is Related to Increased Cytochrome c oxidase Activity in hCOX-2 Tg Mice

**Figure 9:**
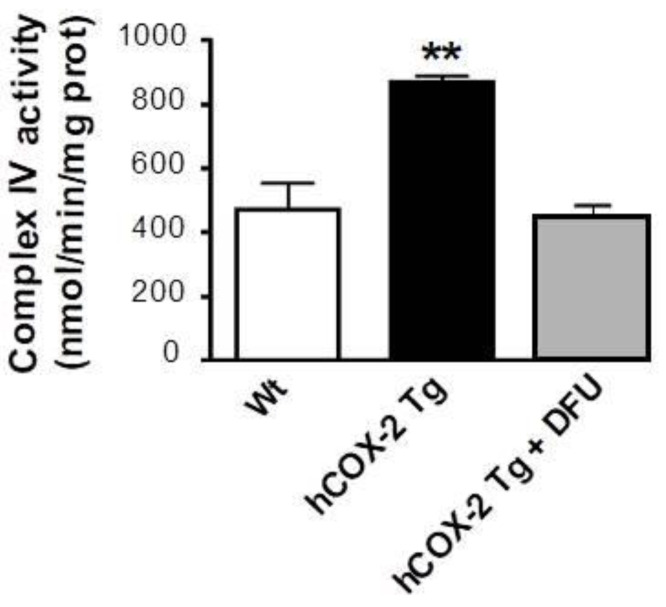
Cytochrome c oxidase activity is elevated in *hCOX-2 Tg* mice. The maximal activity of complex IV was measured in heart lysates *Wt* (*n* = 9), *hCOX-2 Tg* (*n* = 9), and *hCOX-2 Tg* mice treated with the COX-2 inhibitor DFU (*hCOX-2 Tg*+DFU, *n* = 9). Data represent the mean values ± SD; ** *p* < 0.01 *hCOX-2 Tg* mice vs. *Wt* or *hCOX-Tg*+DFU animals.

The maximal activity (Vmax) of respiratory chain complex IV was measured in the heart homogenate from *Wt* and *hCOX-2 Tg* mice. The results showed a significant increase in cytochrome c oxidase activity. In addition, treatment of *hCOX-2-Tg* mice with DFU (COX-2 specific inhibitor) reversed the effects on complex IV activity induced by COX-2-dependent prostaglandins. (Figure 9).

## 3. Discussion

Numerous pro-inflammatory mediators are known to contribute to myocardial dysfunction after reoxygenation of the ischemic myocardium. Among the most important mediators are prostanoids. We previously examined the effects of myocardial expression of hCOX-2 on IRI using mice genetically modified to selectively express hCOX-2 in cardiomyocytes (*hCOX-2 Tg*). Our study demonstrated an increased tolerance to IRI in transgenic mice [12]. The constitutive COX-2 expression resulted in a robust increase in myocardial PGE_2_ levels, eight-fold higher than in *Wt* hearts. This difference is comparable to that detected after viral infection [16], and much lower than in neonatal rat cardiomyocytes after Il-1β treatment [17]. COX-2 is expressed in the infarcted myocardium mainly in the cardiomyocytes, macrophages, vascular endothelium, and endocardium, although the expression is only prominent in the long term in macrophages, focusing on the inflammatory role of the prostanoids. Therefore, our experimental model appears to recapitulate the PC response in the cardiovascular system. To elucidate the molecular mechanisms mediating these cardioprotective effects, we used here a quantitative proteomic approach to identify the protein expression changes in *hCOX-2 Tg* hearts. The main findings in this study indicate that COX-2 induces alterations in mitochondrial respiratory activity that appear to be regulated by specific changes in the mitochondrial proteome.

It is proposed that ischemic PC might initiate signaling that converges on mitochondrial changes resulting in cardioprotection. PC increased the protein abundance of several electron transport chain components, namely complexes III, IV, and V [18]. Accordingly, our results showed that COX-2 expression in cardiomyocytes induces a specific increase in the levels of the subunits of complex IV (Figure 7), which might be one of the mechanisms by which specific cardiomyocyte COX-2 expression generates a cardioprotective phenotype of similar magnitude to that obtained after ischemic PC [12].

We have recently shown that in the liver, under basal conditions, COX-2 has no effect at the mitochondrial level. However, after ischemia/reperfusion injury, mitochondria show a preserved functionality, increased respiratory capacity at the level of complex I and a mitochondrial membrane potential maintained through the OMA-1-regulated OPA1 processing. In this model of COX-2 overexpression in the hepatocyte, we did not detect changes in the amount of mitochondrial proteins or the activity of complex IV [19]. It is known that each tissue has a different metabolic profile and variable energetic demands due to inherently different functions. Therefore, it is reasonable to expect that their mitochondrial respiratory capacity is also different and is related to inter-tissue variations in mitochondrial function, protein composition and morphology [20], as well as in responding to stimuli and damage. Cytochrome c oxidase (COX) activity is very different from organ to organ. For example, inhibiting total COX activity by 80% in liver has a small effect on total respiration, whereas the same degree of inhibition in the heart reduces its respiration rate by 60% [21]. The study of the mitochondrial function of fibers from animals overexpressing COX-2 in hepatocytes also showed an increase in complex IV activity, both under baseline conditions and after ischemia/reperfusion damage to the liver. Thus, COX-2 expression affects mitochondrial function differently according to the differences observed between tissues and stimulus.

The analysis of coordinated protein responses showed decreased glycolysis as one of the differential pathways in *hCOX-2 Tg* mice hearts. Proteins, such as LDHB, PGAM2, GOT1, PKM2 or AK1, whose expressions are decreased in *hCOX-2 Tg* animals, have been identified as potential biomarkers during myocardial ischemia [22]. Moreover, it has been demonstrated that energy metabolism during ischemia is markedly slowed by PC, resulting in reduced accumulation of metabolic products and temporary preservation of ATP stores. Either preservation of ATP or limitation of catabolite accumulation (or both) may be responsible for increased cell viability [23]. Furthermore, intracellular acidosis is an important cause of ischemic contractile dysfunction and myocardial injury. One proton-producing mechanism that could be affected by PC is a diminished glycolytic rate during ischemia [24].

The systems biology analysis also showed that the calcium signaling pathway was upregulated by the constitutive expression of COX-2 through upregulation of VDAC proteins (Figure 5C). Intracellular Ca^2+^ handling in cardiac muscle cells is tightly regulated by a subcellular network involving multiple proteins, pathways and organelles, including mitochondria. It is well established that VDACs form an important permeability pathway for the transfer of Ca^2+^ across the outer mitochondrial membrane. VDAC proteins have essential functions for the survival of cells by mediating the exchange of mitochondrial metabolites and ATP [25]. Ca^2+^ fluxes appear to play a critical role in the control of the contraction–relaxation cycle and in generating eicosanoids that trigger maladaptive changes; however, the preexisting COX-2 metabolites appear to prevent such Ca^2+^ overload, as occurs after PC [26].

An additional role of COX-2 in the mitochondria is its effect on the dynamic supra-organization of the respiratory complexes (supercomplexes or respirasomes) in the inner membrane. Direct interactions of complexes I and III, complexes I and IV, and complexes III and IV have been shown in bovine heart mitochondria [27]. We evaluated the amount of complexes of the OxPhos pathway by BNGE (Figure 7). Immunoblot analysis confirmed the proteomic data and showed a significant increase in complex IV. Furthermore, we found an increase in the levels of all supercomplexes containing cytochrome c oxidase. These results agree with the key role of complex IV in the stoichiometries between large and small supercomplexes that depend on their levels, and they seem to reflect the cellular demand for energy supply via the OxPhos pathway [28]. The increased amount of the complex IV levels seemed to be specific and was not associated with changes in mitochondrial biogenesis or mitochondrial size and fusion.

The activity of cytochrome c oxidase was increased in the hearts of *hCOX-2 Tg* mice according to the variations in the amount of respiratory chain complexes. The enhancement of complex IV activity should improve the efficiency of electron flow from cytochrome c to molecular oxygen, which in turn would better support the inner mitochondrial membrane proton gradient and improve myocardial aerobic respiration [29]. Thus, a significant increase in respiratory capacity and ATP production was found in *hCOX-2 Tg* mice. McGuiness et al. demonstrated that glutamine pretreatment is associated with cardioprotection through an increase in COX-2 expression [30]. It has been shown that glutamine may improve cardiac progenitor cell therapy after myocardial dysfunction by enhancing mitochondrial function [31]. Therefore, our data support the concept that the preconditioned mitochondria or situations that mimic PC, such as after COX-2 expression or glutamine treatment, lead to restored respiration, ATP-generating activity [32] and maintenance of cellular ATP levels to delay myocyte death. This may be the central component of COX-2-dependent cardioprotection.

## 4. Materials and Methods

### 4.1. Chemicals

Antibodies were from Proteintech (Rosemont, IL, USA), Cayman Chemical (Ann Arbor, MI, USA), Abcam (Cambridge, UK), Santa Cruz Biotechnology (Dallas, TX, USA) or Sigma-Aldrich (Merck Life Science S.L.U. Darmstadt, Germany). Reagents were from Roche Diagnostics (Basel, Switzerland) or Sigma (Merck Life Science S.L.U. Darmstadt, Germany). Reagents for electrophoresis were from Life Technologies (Thermo Fisher Scientific, Waltham, MA, USA). The COX-2 inhibitor 5, 5-dimethyl-3(3-fluorophenyl)-4-(4-methylsulfonyl) phenyl-2(5H)-furanone (DFU) was from Merck (Merck Life Science S.L.U. Darmstadt, Germany).

### 4.2. Animal Model

Male *hCOX-2-Tg* mice (25-30g body weight; 3 months) on a B6D2/JRccHsd background were used in this study along with corresponding age-matched *Wt* mice [12]. Only male mice were used in procedures to avoid hormonal modulation of endogenous prostaglandin levels. *hCOX-2 Tg* mice and their corresponding *Wt* littermates were generated by systematic mating of B6D2-Tg (MHC-*PTGS2*)17/Upme Tg mice with B6D2F1/JRccHsd *Wt* mice in our animal house for more than seven generations. In some experiments, mice were treated ip daily for four days with DFU, 5 mg/kg body weight, to inhibit COX-2 [33]. The animals were given diet and water ad libitum. All the experiments were performed following the animal care guidelines of the European Union (2010/63/EU) and approved by the Bioethical Committee from Consejo Superior de Investigaciones Científicas, CSIC.

### 4.3. Isolation of Cardiomyocytes

Ventricular cardiomyocytes were isolated from adult mice following the procedure described previously [34]. Briefly, mice were anesthetized with sodium pentobarbital (100 mg/kg i.p.) and heparinized (4 U/g i.p.). The heart was cannulated via the ascending aorta on a Langendorff perfusion system. Retrograde perfusion was initiated with Ca^2+^-free Tyrode’s solution (130 mM NaCl, 5.4 mM KCl, 0.5 mM MgCl_2_, 25 mM HEPES, 0.4 mM NaH_2_PO_4_, 22 mM glucose, pH = 7.4 adjusted with NaOH) containing 0.2 mM EGTA at room temperature for 2 min and then with the same Tyrode’s solution containing collagenase type II (1 g/L, 3 min) (Worthington Biochemical, Lakewood, NY) and CaCl_2_ (0.1 mM). After that, the heart was removed from the Langendorff system, and the ventricles were cut out into small pieces and mechanically dissociated in the enzymatic solution. The cardiomyocyte cell suspension was then filtered through a nylon mesh (250 μm), pelleted by centrifugation for 4 min at 16× *g* and resuspended in Tyrode’s solution containing 0.5 mM CaCl_2_. Cardiomyocytes were centrifuged as before and suspended in a storage solution containing 1 mM CaCl_2_. Cardiomyocytes were homogenized in a lysis buffer containing 50 mM Tris, 320 mM sucrose, 1 mM DTT, 10 μg/mL leupeptin, 10 μg/mL soybean trypsin inhibitor and 0.2% Nonidet. Extracts were vortex-mixed for 30 min at 4 °C and, after centrifuged for 20 min at 13,000× *g*, the supernatants were stored at −20 °C.

### 4.4. Quantitative High-Throughput Analysis of Proteome by ^18^O Labeling

Peptides and proteins from cardiomyocytes were trypsin-digested using the whole proteome in-gel digestion protocol, followed by ^18^O labeling as previously described [35]. The peptide pools were separated in 24 fractions ranging from pH 3–10 by IEF on a 3100 OFFGel fractionator (Agilent, Santa Clara, CA, USA) using the standard methods for peptides recommended by the manufacturer. The recovered fractions were desalted using OMIX C18 tips (Varian, Inc, Agilent, Santa Clara, CA, USA), and dried down before reverse phase-high performance liquid chromatography (RP-HPLC)-LIT analysis using a Surveyor LC System coupled to a linear ion trap mass spectrometer LTQ (Thermo Fisher Scientific, Waltham, MA, USA). The LTQ was operated in a data-dependent ZoomScan and MS/MS switching mode as previously described [36]. Protein identification was performed using SEQUEST algorithm (Bioworks 3.2 package, Thermo Fisher Scientific, Waltham, MA, USA). MS/MS raw files were searched against a Rat/Mouse Swissprot database supplemented with the sequence of bovine and porcine trypsin. SEQUEST results were analyzed using the probability ratio method [37], and discovery rates (FDR) of peptide identifications were calculated as previously described [38]. Peptide identification and quantification were performed as previously described [35,39]. Statistical significance of protein abundance changes was assayed by controlling the FDR, with an FDR less than 0.05 considered to be significant. Threshold-free analysis of coordinated protein responses was performed using the SBT model, as described [14].

### 4.5. Functional Classification

Predictive functional classification of differentially expressed proteins was performed using ingenuity pathway analysis (Ingenuity Systems, Redwood City, CA, USA). Pathway analysis, biological functions and sub-cellular localization were predicted to reveal biologically relevant proteins and associated networks. Fisher’s exact test was used to calculate the *p* value determining their statistical significance.

### 4.6. DNA Isolation and qPCR for Mitochondrial to Genomic DNA Ratio

Total DNA was isolated from small pieces of frozen tissues (~1 cm^2^). The pieces were homogenized in 100 μL of Buffer I (10 mM Tris-HCl, pH 7.0, 10 mM MgCl_2_, 0.5% (*v*/*v*) Triton X-100) in a small glass-potter manually. The homogenate was transferred to a 1.5 mL tube and 5 U/μL of RNase was added. The tubes were incubated at 37 °C for 30 min. Next, 300 μL of lysis buffer (2% (*w*/*v*) cetyl trimethyl ammonium bromide (CTAB), 100 mM Tris-HCl, pH 8.0, 20 mM EDTA, 1.4 M NaCl) plus 2% (*v*/*v*) β-mercaptoethanol was added. A brief vortex was applied, and the tubes were incubated at 65 °C for 30 min. After this time, a volume of chloroform–isoamyl alcohol (24:1) was added, the tubes were shaken until the combination of the 2 phases was achieved and centrifuged at 16,000× *g* for 10 min at room temperature. The upper phase was then collected in new tubes, and a volume of isopropanol was added. The tubes were kept at −20 °C for at least 1 h and then centrifuged at 16,000× *g* for 25 min at 4 °C. The supernatant was discarded and the DNA pellet was washed twice with 70% EtOH, dried at room temperature and resuspended in 10 mM Tris/0.1 mM EDTA. DNA quantification was performed and diluted to a final concentration of 10 ng/mL. The *CytB* gene as mitochondrial DNA (mtDNA) marker and *ApoB* gene as nuclear DNA (nDNA) were amplified by qPCR with Power SYBR Green Master Mix (Thermo Fisher Scientific, Waltham, MA, USA). The primer sets used were: *Cytb*-forward 5′-GCTTTCCACTTCATCTTACCATTTA-3′; *Cytb*-reverse 5′-TGTTGGGTTGTTTGATCCTG-3′; *ApoB*-forward: 5′-CGTGGGCTCCAGCATTCTA-3′; *ApoB*-reverse 5′-TCACCAGTCATTTCTGCCTTTG-3′. The used protocol was 20 s at 95 °C, then 40 cycles of 1 s at 95 °C, followed by 20 s at 60 °C, and a final melt curve stage of 1 s at 95 °C, 20 s at 60 °C, and 1 s at 95 °C. Each sample was run in duplicate, and mtDNA was normalized to nDNA. The biological replicates were then averaged, and fold induction was determined in a 2^−∆∆Ct^-based fold-change calculation.

### 4.7. Transmission Electron Microscopy (TEM)

For TEM, epoxy resin inclusion was performed by fixing hearts with 2% glutaraldehyde, in 0.1 M sodium cacodylate pH 7.2, washed with cacodylate buffer containing 0.1 M sucrose, and post-fixed with 1% osmium tetroxide in phosphate buffer. After washing with water and dehydration with ethanol, samples were embedded in epoxy resin. Ultrathin slides (60 nm) were finally stained with 2% uranyl acetate before viewing by TEM using a Jeol JEM1010 microscope (Jeol, Akishima, Tokio, Japan) at 60 kV. Images were acquired with a digital camera AMTR×80 with Olympus Image Analysis Software (Olympus, Shinjuku, Tokio, Japan). Quantitative analyses of mitochondrial size and density were carried out at a magnification of ×1200, ×3000 and ×6000. An average of fifteen visual fields was evaluated for each mouse heart.

### 4.8. RNA Isolation

Total RNA from the heart was extracted by using TRIzol reagent (Thermo Fisher Scientific, Waltham, MA USA). First strand cDNA was synthesized from 1 μg of total RNA using random hexamer and expand reverse transcriptase (Roche Basel, Switzerland), as used as a template for real-time PCR with FastStart Universal SYBR Green Master (Roche). The qPCR reaction was performed in 20 µL with primers for *Mtco1* (forward: ACCCAATATCAGACACCTCTCTTTGT; reverse: GACGGCTGTAATTAGTACGGATCATAC), *Mtco2* (forward: AACCAAGCTACAGTGACATCAAACC, reverse: GCATTGGCCATAGAATAGACCTG) and *Gapdh* as housekeeping gene (forward: GTATTGGGCGCCTGGTCAC, reverse: AATCTCCACTTTGCCACTGCA). The used protocol was identical for all primer sets: 20 s at 95 °C, then 40 cycles of 3 s at 95 °C, and 30 s at 60 °C. The raw (i.e., not baseline-corrected) PCR data were exported into an Excel datasheet and analyzed using LinRegPCR 2018.0 software (Amsterdam UMC, Amsterdam, The Netherlands) [40].

### 4.9. Preparation of Mouse Heart Mitochondria

Mouse cardiac mitochondria were isolated by differential centrifugation. Briefly, hearts were homogenized in cold isolation buffer A (210 mM mannitol, 70 mM sucrose, 50 mM Tris-HCl, pH 7.4, and 10 mM EDTA). The homogenate was centrifuged at 1000× *g* for 5 min, and the resulting pellet was again washed with cold medium A and centrifuged under the same conditions. The supernatant fractions were collected and centrifuged at 7000× *g* for 10 min. The pellet was resuspended in ice-cold isolation medium B (225 mM mannitol, 75 mM sucrose, 10 mM Tris-HCl, pH 7.4, and 0.1 mM EDTA), and a new series of centrifugations (1000× *g* and 7000× *g*) was performed. The crude mitochondrial pellet was resuspended in a minimum volume of isolation medium B to obtain a mitochondrial concentration of between 9 and 14 mg/mL. Protein concentration was measured by the Lowry method using BSA as standard.

### 4.10. Analysis of the Respirasome Composition by Blue-Native Electrophoresis (BNE) and Western Blotting (BNGE)

Isolated mitochondria were centrifuged at 7000× *g* for 10 min at 4 °C. The mitochondria pellet was resuspended in an appropriate volume of resuspension buffer (1 M 6-aminohexanoic acid, 50 mM Bis-Tris (HCl), pH 7.0) to a final mitochondrial concentration of 10 μg/μL. Then, 100 μg of mitochondria was incubated 5 min on ice with 10% digitonin (10% (*w*/*v*) digitonin in 50 mM NaCl, 50 mM imidazole, 5 mM 6-aminohexanoic acid, 4 mM PMSF), and then centrifuged at maximum speed for 30 min at 4 °C. The supernatant was collected in a new tube, and blue sample buffer (5% (*w*/*v*) blue G dye, 1 M 6-aminohexanoic acid) was added (20% of the final volume). Samples were loaded in the XCellSureLock Mini-Cell Electrophoresis System (Thermo Fisher Scientific, Waltham, MA, USA); dark blue cathode buffer (20% (*w*/*v*) coomassie brilliant blue G250 in anode buffer) was loaded on the inside and anode buffer (NativePAGE Running Buffer 20×, Thermo Fisher Scientific, Waltham, MA, USA) in the outside. The gel was run 30 min at 150 V at 4 °C, then the dark blue cathode buffer was replaced with light blue cathode buffer (1/10 dilution dark blue cathode buffer: anode buffer), and run for an additional 60 to 150 min at 250 V at 4 °C. At the end of the run, the gel was stained with blueSafe (NZYTech, Lisbon, Portugal) and scanned or transferred to a PVDF membrane for immunoblotting assays. The complexes were identified with specific antibodies against complex I (NDUFA5 subunit; Proteintech Rosemont, IL, USA, No. 16640-1-Ap), complex III (ubiquinol-cytochrome c reductase core protein II; Sigma, GW22153A Merck Life Science S.L.U. Darmstadt, Germany) and complex IV (cytochrome c oxidase subunit II; sc-23983 Santa Cruz Biotechnology, Dallas, TX, USA). After incubation with the corresponding horseradish peroxidase conjugated secondary antibody, blots were developed by the ECL protocol. Target protein band densities were normalized by calculating the ratio to the corresponding densities of TOMM20 protein (Sigma, WH0009804M1 Merck Life Science S.L.U. Darmstadt, Germany). Different exposure times were performed on each blot to ensure the linearity of the band intensities. Densitometric analysis was expressed in arbitrary units.

### 4.11. Western Blot Analysis

Heart tissue was homogenized in a RIPA lysis buffer (50 mM Tris-HCl pH 8, 150 mM NaCl, 1% NP40, 0.5% sodium deoxycholate, 0.1% SDS, supplemented with protease inhibitor cocktail tablets (Roche Basel, Switzerland)) at 4 °C. After centrifuging for 30 min at 14,000× *g*, the supernatants were stored at −80 °C. For Western blot analysis, samples were boiled for 5 min in Laemmli sample buffer (5% SDS, 10% glycerol, 25 mM Tris-HCl pH: 6.8, 10 mM DTT, 0.01% bromophenol blue), and equal amounts of protein (30 μg) were separated on 8-15% SDS-polyacrylamide electrophoresis gels (SDS-PAGE). The relative amounts of each protein were determined with the following polyclonal or monoclonal antibodies: COX-1 (sc-1752 Santa Cruz Biotechnology, Dallas, TX, USA), COX-2 (160112Cayman Chemical Ann Arbor, Michigan USA); mitofusin 2 (ab56889Abcam Cambridge, UK,); TOMM20 (Sigma, WH0009804M1 Merck Life Science S.L.U. Darmstadt, Germany); β-actin (Sigma, A2066 Merck Life Science S.L.U. Darmstadt, Germany). After incubation with the corresponding horseradish peroxidase conjugated secondary antibody, blots were developed by the ECL protocol (GE Healthcare, Chalfont St Giles, UK). Protein band densities were normalized to β-actin. Densitometric analysis was carried out with Image J software and expressed in arbitrary units.

### 4.12. Mitochondrial Function

Mitochondrial oxygen consumption was monitored in isolated saponin permeabilized fibers from freshly excised hearts at 37 °C in a 1 mL thermostatically controlled chamber equipped with a Clark oxygen electrode (Oxy 1, Hansatech Narborough Rd, Pentney UK) in respiration buffer (75 mM mannitol, 25 mM sucrose, 100 mM KCl, 10 mM Tris phosphate, 10 mM Tris-HCl pH 7.4, 50 µM EDTA). The respiratory rate was expressed in nmolO_2_/min and mg of dry fiber. ATP content was determined using the ATP Bioluminescence Assay Kit HS II (Roche Basel Switzerland) according to the manufacturer’s instructions. Briefly, the frozen apex region of each heart was mechanically disrupted in cold lysis buffer, incubated for 2 min at 95 °C and centrifuged at 14,000× *g* for 3 min. Then, 50 µL of the pooled supernatant was utilized to determine ATP levels using ATP stock provided by the kit as standard. Data were normalized to the protein concentration.

### 4.13. Measurement of Complex IV Activity

Hearts were mechanically disrupted using a Dounce homogenizer in isotonic buffer (10% *w*/*v*) (225 mM mannitol, 75 mM sucrose, 10 mM Tris-HCl, 0.1 mM EDTA, pH 7.2) supplemented with protease inhibitors (Roche Basel Switzerland) following the manufacturer’s indication. The homogenate was centrifuged for 20 min at 650× *g*, and supernatants were used for cytochrome c oxidase analysis. Mitochondrial cytochrome c oxidase activity, measured at 37 °C by the changes in absorption at 550 nm, was determined in heart homogenates using standard spectrophotometric methods using reduced cytochrome c as substrate [20]. The extinction coefficient used for cytochrome c was 18.5 (mM)^−1^ cm^−1^. The protein concentration was determined by the Lowry method.

### 4.14. Statistical Analysis

Statistical analyses were performed using the SPSS (IBM Corp. Released 2020. IBM SPSS Statistics for Windows, Version 27.0, Armonk, NY: IBM Corp). Student’s *t* test was applied whenever necessary, and statistical analysis of the differences between groups was performed by one-way ANOVA followed by Tukey’s test. A *p* value of less than 0.05 was considered to be significant.

## 5. Conclusions

Our study is the first to demonstrate that COX-2 in cardiomyocytes can increase specific complex subunits of the OxPhos pathway, improving the respiratory capacity and ATP production of the mitochondria. Furthermore, COX-2 could be involved in metabolic changes reminiscent of those achieved after PC. COX-2 is an obligatory mediator of the late phase of ischemic preconditioning. Consequently, we reasoned that one of the roles of COX-2 during this phase involves an alteration in the assembly of mitochondrial supercomplexes that might modulate the mitochondria fitness for synthesizing ATP, preventing the generation of harmful metabolites released after reoxygenation.

## Figures and Tables

**Table 1:** Differentially regulated proteins in response to cyclooxygenase-2 constitutive expression sorted according to fold change. Standardized variable at the protein level was calculated as previously described [13].

Accession	Gene	Description	Subcellular Localization	Zq	FDRq	Total Peptide Count
Q7M6Y9	*Heatr7b2*	Uncharacterized protein		↑10.31	0	1
P00405	*Mtco2*	Cytochrome c oxidase subunit 2	Mitochondrion inner membrane	↑6.53	7.70 × 10^−9^	6
Q8R0F8	*Fahd1*	Fumaryl acetoacetate hydrolase domain-containing protein 1	Mitochondrion	↑6.16	8.33 × 10^−8^	1
P19783	*Cox4i1*	Cytochrome c oxidase subunit 4 isoform 1	Mitochondrion inner membrane	↑6.03	1.29 × 10^−7^	6
P12787	*Cox5a*	Cytochrome c oxidase subunit 5A	Mitochondrion inner membrane	↑5.96	1.95 × 10^−7^	4
Q9D8B4	*Ndufa11*	NADH dehydrogenase [ubiquinone] 1 alpha subcomplex subunit 11	Mitochondrion inner membrane	↑5.91	1.99 × 10^−7^	1
Q60932	*Vdac1*	Voltage-dependent anion-selective channel protein 1	Mitochondrion outer membrane	↑5.37	4.63 × 10^−6^	7
Q6AYI5	*Gpatc2*	G patch domain containing 2		↑5.28	7.64 × 10^−6^	1
P19536	*Cox5b*	Cytochrome c oxidase subunit 5B	Mitochondrion inner membrane	↑5.20	8.58 × 10^−6^	1
Q8R2U7	*Lrrc42*	Leucine-rich repeat-containing protein 42		↑4.97	2.89 × 10^−5^	1
P56392	*Cox7a1*	Cytochrome c oxidase subunit 7A1	Mitochondrion inner membrane	↑4.66	0.0001	1
P00397	*Mtco1*	Cytochrome c oxidase subunit 1	Mitochondrion inner membrane	↑4.55	0.0002	1
Q60930	*Vdac2*	Voltage-dependent anion-selective channel protein 2	Mitochondrion outer membrane	↑4.42	0.0003	4
A2AL77	*Asph*	Aspartate-beta-hydroxylase		↑4.35	0.0004	1
Q99KW3	*Triobp*	TRIO and F-actin-binding protein	Cytoplasm	↑4.12	0.001	1
Q60931	*Vdac3*	Voltage-dependent anion-selective channel protein 3	Mitochondrion outer membrane	↑4.11	0.001	3
Q61292	*Lamb2*	Laminin subunit beta-2	Extracellular matrix	↑3.75	0.004	1
Q791V5	*Mtch2*	Mitochondrial carrier homolog 2	Mitochondrion inner membrane	↑3.58	0.007	1
Q9ES82	*Popdc2*	Popeye domain-containing protein 2	Membrane	↑3.33	0.018	1
Q9CRY7	*Gdpd1*	Glycerophosphodiester phosphodiesterase domain-containing protein 1	Membrane	↑3.31	0.018	1
Q9D1R1	*Tmem126b*	Transmembrane protein 126B	Membrane	↑3.29	0.019	1
D3ZX78	*Vom2r16*	Protein Vom2r16	Membrane	↑3.28	0.019	1
Q61941	*Nnt*	NAD(P) transhydrogenase	Mitochondrion inner membrane	↓2.99	0.046	12
Q9R0Y5	*Ak1*	Adenylate kinase isoenzyme 1	Cytoplasm	↓3.20	0.025	4
Q3UJH8	*Got1*	Aspartate aminotransferase	Cytoplasm	↓3.94	0.002	1
Q8K3Q4	*Actn2*	Actinin alpha 2		↓4.73	8.16 × 10^−5^	1
Q3V0K9	*Pls1*	Plastin-1	Cytoplasm	↓15.88	0	1
Q6TUF7	*LRRGT00087*			↓25.09	0	1

## Data Availability

All the proteins identified in the proteomic analysis have been deposited in open access at Digital CSIC, which can be accessed through the following link: https://doi.org/10.20350/digitalCSIC/14752 (accessed 29 September 2022).
